# Supplementary material for: Are carbon emissions trading and green financial instruments synergistic? -Comprehensive quantitative research based on content analysis
Source: PLoS One. 2024 Mar 7;19(3):e0298601. doi: 10.1371/journal.pone.0298601 (PMC10919601; doi:10.1371/journal.pone.0298601)
Supplement: S1 Data — (ZIP) [file pone.0298601.s001.zip › Data/地级市Z_F工作报告.zip!/║■─╧/╓Ω╓▐╩╨/└┤╫╘╣1⁄2╓┌║┼í╛┬φ┐╦╩2╛▌═°í┐_2012.docx]

各位代表：

　　现在，我代表市人民政府向大会作政府工作报告，请予审议，并请各位市政协委员和其他列席同志提出意见。

　　锐意进取：努力实现“十二五”良好开局

　　2011年是“十二五”的开局之年，是建市60周年的喜庆之年，也是经济社会发展取得显著成效的一年。一年来，我们在市委的坚强领导下，围绕“保二争一、科学跨越”战略目标，按照“保增速、保提质、保民生”的总体要求，加快转变发展方式，积极推进“两型”建设，深入打好“三大战役”，大力实施“四大战略”，全面建设“四个株洲”，完成或超额完成市十三届人大四次会议确定的各项目标任务。全市GDP达到1550亿元，增长13.6%；财政收入达到175.3亿元，增长33.9%；实现全社会固定资产投资855亿元，增长36.6%；城镇居民人均可支配收入达到22490元，农民人均纯收入达到9040元，分别增长14.5%、18%；万元GDP能耗下降3.5%，主要污染物排放总量下降3%。

　　发展质量明显提升。新型工业化加速推进。实现工业增加值830亿元，增长16.5%，其中规模工业增加值730亿元，增长19%，新型工业化考核连续五年获全省一等奖。“5115”企业总产值达780亿元，增长25%，年销售收入过100亿元的企业3家，过50亿元的企业4家。中小企业实现规模工业增加值500亿元，增长20.3%；中小企业服务超市、创业成长俱乐部、产业联盟等服务载体得到发展。攸县煤电一体化、中航高精传动、中国五矿精密工具产业园启动建设，株洲县风力发电项目正式落户，时代电气IGBT产业化、株洲通用机场、南车时代新能源客车基地、时代风电等项目建设进展顺利，北汽集团株洲基地一期、南车株机不锈钢城轨车辆项目建成投产。继续打好园区攻坚战役，启动园区建设项目169个，完成投资270亿元，实现园区技工贸总收入1600亿元，增长22.8%；高新技术产业产值达960亿元，增长22%；轨道交通、汽车、航空、服饰、陶瓷等产业集群不断发展壮大。农业现代化稳步实施。完成农业增加值130亿元，增长4%。粮食总产量突破190万吨，连续8年实现丰产丰收。农业机械化率达到62.1%。生猪养殖规模化率超过60%。农产品加工业总产值达182亿元，增长19%。醴陵市跻身全国经济百强县，攸县稳居全省县域经济十强县。现代服务业全面提升。实现社会消费品零售总额505亿元，增长18.2%。红星美凯龙、沃尔玛等项目相继启动。继续打好旅游升温战役，全年实现旅游总人数1625万人次，增长33.2%；旅游总收入109.6亿元，增长33.6%；神农谷、酒仙湖成功创建为国家4A级旅游景区，全国首家红军标语博物馆建成开馆；国际品牌酒店希尔顿、万豪相继开工建设。芦淞服饰城综合提质改造工程全面完成，获评“中国（中南）服装第一街”。商品房销售面积640万平方米，增长24.7%。金融机构年末存款余额达1300亿元，新增163.4亿元，增长12%；贷款余额670亿元，新增106亿元，增长18.5%。唐人神和旗滨两家股份有限公司成功上市，上市公司达到9家。铜塘湾新港区一期主体工程基本完工，南车株洲物流基地、湘江金属物流城建设取得实质性进展。中国驰名商标和著名商标分别达到20件和155件，获评“国家商标战略实施示范城市”。非公经济占GDP的比重达到55%。招商引资态势强劲。共引进项目660个，到位资金总额突破300亿元，增长100%。上海宝钢集团、中国水利水电建设集团等知名企业进驻株洲，在株央企达到17家，世界500强企业达到10家。外贸结构进一步优化，加工贸易比重大幅提高，机电产品出口完成5.06亿美元，增长79.3%，完成外贸进出口总额18.4亿美元，增长24%。

　　发展后劲明显增强。重大基础设施建设全面推进。在建高速公路257公里，改建干线公路275.6公里，开工建设干线公路405公里，改造农村公路1180公里。浏醴、分炎、炎睦、醴茶、炎汝、茶界等高速公路建设扎实推进，株洲西互通改造工程、S315龙下至攸县段、芷渌公路竣工通车，攸县广志村至山门洪段、S322炎陵牛岗排至资兴深坳段、S211株洲段、G106炎陵至槽里段基本完工。衡茶吉铁路、沪昆高铁、长株潭城际铁路建设加速推进。湘江航道疏浚一期工程进展顺利，洮水水库成功下闸蓄水。湘江六桥、东环北路、荷塘大道、航空大道开工建设。城市品质品位继续提升。创建国家环保模范城市已通过省预验收，创建全国文明城市获得提名资格。神农城一、二期，湘江风光带河西城区段建成开放。响石广场、向阳广场、株醴路、体育路、钻石路等改造工程全面竣工。城区主次干道无障碍设施改造全面完成。建成自行车专用车道和公共自行车租赁系统，已投放自行车1万辆，被列为全国“城市步行和自行车交通系统示范项目”试点城市。新增绿地面积32.9公顷，城市绿化覆盖率提高到50%。美化建筑33栋，改造小街小巷60条。亮化道路路灯13条，新建小街小巷路灯60条，城区无灯路段全部消除。数字株洲建成视频融合平台，平安城市、智能交通、数字城管三大平台全面整合。株洲、醴陵、攸县分别获评湖南“最干净”地级市、县级市和县城，攸县城乡环境同治经验在全国推广，获评“全国生态文明先进县”。农村基础设施不断改善。建成乡村通畅工程1000公里、农村乡镇客运站8个。投资6.43亿元，完成各类水利工程建设2.25万余处，治理病险水库16座。新建沼气池7548口，解决了农村12.76万人饮水不安全问题。云田、松西子、仙庾等新农村示范点建设步伐加快。森林覆盖率提高到61.5%，炎陵县被评为“国家林下经济示范县”。

　　发展活力明显提高。“两型”改革继续深化。按照“资源资产化、资产资本化、资本证券化”的思路，“6+2”投融资公司着力提升经营水平，城发集团、高科集团分别成功发行15亿元、10亿元企业债券，为基础设施建设提供了强大资金支持。统筹城乡发展改革形成了高塘等一批典型模式，完成了53个村改社区工作，土地利用集约化、产业发展集聚化程度明显提高。进一步规范农村土地经营流转，地票交易实现常态化，连续13年实现耕地占补平衡，集体林权制度改革全面完成。率先在全省开展排污权交易试点工作，全面启动节能减排“三百工程”。示范区建设纵深推进。云龙示范区华强方特欢乐世界建成开园，职教大学城已有7所院校入园，2所院校建成开学，云龙大道基本建成，华强路、云峰大道（复线）、迎宾大道建成通车，“生态城、科教城、旅游城”初具规模。天易示范区高科汽配园项目一期基本完工，栗雨中央商务区、新马片区开发稳步推进，“两型”产业集聚区逐渐形成。清水塘循环经济示范区战略性改造工程正式启动，6个废水废渣处理项目相继实施。“两型”环境加速构建。“一江四港”环境综合治理工程全面启动，三角叉水系改造、建宁港截污干管建设全面完成，董家塅和龙泉污水处理厂污泥处置中心、清水塘重金属污水处理厂投入运行，霞湾港整治工程、白石港污水处理项目进入实施阶段。南郊垃圾处理场提质改造工程全面竣工，城镇生活垃圾处理设施建设进展顺利。10个限期治理大气污染项目全部完成，39家污染企业关闭或搬迁。湘江株洲段水质保持Ⅲ类标准，市区集中式饮用水源水质达标率达100%。

　　民生事业明显改善。社会保障不断加强。新增城镇就业5.78万人，新增农村劳动力转移就业7.48万人，实现城镇“零就业家庭”动态就业援助100%，就业创业指导中心建设正式启动。养老医疗保险制度实现城乡全覆盖，企业养老保险新增参保4.66万人，新型农村养老保险参保98.17万人，城镇居民医疗保险参保75.26万人，城镇医疗保险覆盖率稳定在95%以上，新型农村合作医疗参合率达到96.67%。城区最低工资标准由每月800元提高到1020元；城区城镇居民生活最低保障线标准由300元/月提高到330元/月，月人均补差达220元；在全省率先推行市区城乡居民最低生活保障统筹并轨，促进了城乡低保一体化；启动低收入群体价格临时补贴联动机制，困难家庭生活得到有效保障；市儿童社会福利院和流浪未成年人救助保护中心全面竣工。改造农贸市场12个，建成无公害蔬菜基地10300亩。保障性安居工程完成投资13.2亿元，新增廉租房6667套、公租房5585套，改造棚户区8050户；改造农村危房4831户，改造国有林场危旧房935户。住房公积金支持居民购买住房面积102万平方米。社会事业全面发展。成功研制出我国首列中低速磁悬浮列车；建成全国首座电动公交城；争取部省级科技项目90项，其中新增国家863计划重点项目5项，新建省级重点实验室6个；第八次获评“全国科技进步先进城市”；丁荣军当选中国工程院院士。改扩建城区义务教育学校13所，新增学位4544个，建成合格学校46所，资助贫困学生5.02万人；全面完成城区中小学塑胶运动场建设；建宁国际实验学校开工建设；被国家确定为“地方政府促进高等职业教育发展综合改革试点城市”。建市60周年系列活动精彩纷呈，首届湖南合唱节唱响株洲，高水平承办了湖南国际旅游节、辛卯年省长公祭炎帝陵和海峡两岸炎帝神农文化祭活动；建成乡镇综合文化站46个，农家书屋854个。成功举办市第四届大众体育运动会，全民健身运动深入推进。公立医院改革试点工作稳步推进，基本药物制度全面实施，市中心医院正式挂牌，20个社区卫生服务中心建成投入使用，基本建成市区公共卫生和公立医院信息系统。其他工作很有成效。大力开展食品药品安全专项行动，全市未发生一起重大食品药品安全事故。荣获“全国双拥模范城市”五连冠。被授予“全国人防体系建设和管理先进单位”。坚持计划生育基本国策，再次获评“全省人口计生工作模范市”，成为“全国人口计生综合改革示范市”。应急管理机制不断完善，突发事件应对能力明显提高，安全生产形势持续好转，信访形势平稳可控，社会大局和谐稳定。民族宗教、妇女儿童、外事侨务、审计、广播电视、气象、水文、市志、档案、扶贫、助残、移民、老干、对口支援、民兵预备役等工作都有新进步。

　　政府自身建设进一步加强。认真执行人大及其常委会决议、决定，自觉接受人大、政协和社会各界监督，共办结人大代表建议289件，政协委员提案274件，办结率、见面率、满意率均为100%。深入推进项目审批代理制、限时办结制、项目代建制等行政管理体制改革，行政审批效率继续提高。高度重视民主监督，畅通群众诉求渠道，政务公开力度不断加大。12345市长热线处理市民意见、投诉等事项55239件，办结率98.8%。加大审计监督、行政监察和廉政建设力度，行政效能和服务水平都有了明显提升。

　　各位代表，过去的一年，是充满机遇、充满挑战的一年，是克难奋进、富有成效的一年。这一年，我们紧扣加快发展第一要务，积极应对国内外复杂多变的经济形势，经济发展稳步增长，城乡面貌日新月异，综合实力稳步提升；我们紧扣转变方式第一主题，着力优化产业结构，“两型”建设明显加快，发展质量不断提高；我们紧扣改善民生第一责任，坚持不懈为民办实事，努力解决人民群众最直接、最关心、最现实的利益问题，人民生活更加幸福。我们真切地体会到，做好政府工作，必须坚持解放思想，以思想观念的大变革，推动科学发展的大跨越；必须坚持以人为本，以民生事业的大投入，带来群众生活的大变样；必须坚持依法行政，以服务质量的大提升，促进各项事业的大进步。

　　发展的成绩来之不易。这是市委坚强领导的结果，是市人大和市政协监督、支持的结果，是各民主党派、工商联、无党派人士、各人民团体参政议政的结果，汇聚了各位代表和委员的智慧力量，凝聚了全市人民的辛勤汗水。在此，我代表市人民政府，向各位人大代表和政协委员，向驻株部队官兵和全市人民，向关心、支持、参与株洲建设的各界人士，致以诚挚的谢意和崇高的敬意！

　　在总结成绩的同时，我们也清醒地看到，我市经济社会发展中还存在不少困难和问题，主要是：经济总量还不够大，产业结构还不够优，转变发展方式、加快发展速度、提升发展质量的任务依然繁重；资源环境约束趋紧，要素制约依然突出，推动“两型”社会改革建设的体制机制还有待进一步完善；城乡差距仍然较大，统筹城乡发展的任务艰巨，农民增收渠道有待拓宽，新农村建设和小城镇发展需要进一步加强；社会管理和公共服务体系还不够完善，社会矛盾还比较多，一些群众的利益诉求还不能得到很好的满足；政府自身建设需要继续加强，发展环境有待进一步优化；等等。这些问题，我们将始终保持清醒的头脑，以科学务实的态度，坚决有力的措施，切实加以解决。

　　坚定信心：强力保持快速发展态势

　　2012年是本届政府的收官之年，也是实现“十二五”宏伟目标的攻坚之年。当前，中央“稳中求进”的总基调已经确立，国内经济将保持平稳较快发展。全省“四化两型”战略纵深推进，“两个加快”、“两个率先”已成共识。我市经济发展的基础更加扎实，发展的环境更加宽松，发展的合力更加强劲。我们将努力把握机遇，应对挑战，珍惜来之不易的良好发展态势，奋勇前行，推动经济社会又好又快发展，不负人民群众的殷切期待，不负本届政府的历史使命。

　　2012年政府工作的指导思想是：深入贯彻落实科学发展观，紧扣加快转变发展方式主线，全面推进“四化两型”建设，坚持稳中求进，突出“三个加快”，加快发展速度，加快产业转型，加快改善民生，办实事，抓落实，以优异成绩迎接党的十八大召开。

　　今年全市经济社会发展的主要预期目标是：GDP增长13%；财政总收入和一般预算收入增长15%；全社会固定资产投资增长30%；社会消费品零售总额增长18%；城镇居民人均可支配收入和农民人均纯收入分别增长13%和15%；万元GDP能耗下降3.5%以上，主要污染物排放总量削减不低于省下达目标；城镇登记失业率控制在4.5%以内；人口自然增长率控制在7.5‰以内。同时，集中力量突出抓好十大产业项目、十大基础工程和十大民生实事。

　　十大产业项目：1.突出IGBT和中低速磁悬浮等重大项目，加快建设轨道交通千亿产业园。2.突出高精传动、通用机场、轻型飞机和通用发动机等重大项目，加快建设国家航空高技术产业基地。3.突出精密工具产业园，加快建设有色金属新材料精深加工基地。4.突出发动机、轿车生产线等重大项目，加快北汽二工厂建设进度。5.突出芦淞市场群的提质改造，加快服饰产业园建设。6.突出陶瓷艺术城等重大项目，加快建设醴陵陶瓷产业园。7.推进攸县煤电一体化项目建设，完成电厂厂房一期工程。8.推进方特梦幻王国和动漫影视基地建设，基本建成华强文化科技产业园。9.推进旗滨集团整体搬迁改造升级，提升现代建材产业水平。10.加快南车株洲物流基地、湘江金属物流城建设，打造中部地区现代物流行业旗舰。

　　十大基础工程：1.基本建成神农大剧院和神农艺术中心，打造全球华人炎帝文化景观中心。2.启动湘江风光带河东景观工程建设，打造“东方莱茵河”。3.推进“一江四港”综合整治，加快形成城市水生态系统。4.加快湘江六桥建设，完善主城区交通体系。5.加快航空大道、荷塘大道、神农大道、田心大道建设，建成云龙大道，完善中环大道，努力构建“一体三极”格局。6.加快醴茶、浏醴、炎睦、炎汝、茶界、分炎高速公路建设，完善市域2小时高速通勤圈。7.加快职教大学城图书馆和科技馆建设，打造株洲智慧谷。8.启动城市防洪排渍新建、改造工程，提升城市防洪排渍能力。9.推进铜塘湾港区和湘江航道疏浚一、二期工程建设，提升湘江港口吞吐能力。10.抓好霞湾港污染治理、大湖治理、清水湖综合开发项目，加快推进清水塘重金属污染治理。

　　十大民生实事：1.积极扩大就业。新增城镇就业5万人、失业人员再就业2.6万人、“零就业家庭”动态援助100%、新增农村劳动力转移就业5万人。2.缓解就医难题。深入推进公立医院改革试点工作，新型农村合作医疗参合率巩固在95%以上，新农合政策范围内住院费用平均补偿率达到70%；城镇居民医疗保险住院补偿率稳定在70%以上。3.改善就学条件。新建、扩建义务教育学校13所，建成义务教育合格学校23所；资助家庭贫困学生2.2万人；完成城区基础教育扩容提质工程项目7个。4.加强住房保障。新增廉租房4000套，新增廉租住房租赁补贴家庭700户；改造国有林场和农垦林场危旧房1071户；改造农村危房2000户；新增住房公积金建制人数1.5万人。5.完善社保体系。稳定新型农村养老保险参保率85%；新增企业养老保险参保人数4万人；稳定城镇居民医疗保险参保人数69万人。6.保护生态环境。加大“一江四港”治理力度，确保湘江株洲段保持国家Ⅲ类以上水质；加大清洁能源推广和大气污染综合整治，确保空气质量达到二级；加大餐饮、建筑垃圾处理力度，基本建成生活垃圾焚烧发电厂，力争城区生活垃圾无害化处理率达到100%。7.创建平安株洲。实现临街门店、学校、居民小区电子监控系统全覆盖，重点单位、要害部位技防设施安装联网率达到100%。8.加强特殊人群管理。建立市特殊人群管理服务中心，指导全市特殊人群的管理服务工作。9.加强社会救助。将城区城镇居民生活最低保障线标准提高到370元/月；新建和改扩建农村敬老院4所；为困难群众实施法律援助案件1500件。10.保障食品药品安全。健全食品药品质量安全监管体系，搭建“数字食安”监管云平台，建设食品检验检测中心，确保各类食品抽检平均合格率达到95%以上；建设高标准无公害蔬菜基地7000亩；蔬菜农药残留超标率控制在6%以内；米粉企业持证率达到100%；基本药物目录考核品种质量合格率达到98%以上；新解决12万农村群众饮水不安全问题。

　　真抓实干：全力完成今年目标任务

　　我们将围绕政府工作指导思想和发展目标，加快发展速度，加快产业转型，加快改善民生，突出抓好以下五个方面的工作：

　　（一）突出产业转型，提升发展质量

　　优化产业结构。继续实施“6+1+4”产业发展行动计划，改造提升交通装备制造、有色金属深加工、化工、服饰、陶瓷等传统优势产业，发展壮大新能源、新材料、生物医药和健康食品等战略性新兴产业，加快建设“四城三基地”。以规模化、集约化、机械化为重点，加快发展现代农业，抓紧实施粮食增产高效工程和农产品加工业振兴计划，力争培育农产品加工龙头企业10家以上，实现总产值200亿元；组建神农粮油集团，打造现代农业旗舰；启动中南农产品交易（物流）中心、中南粮油交易中心建设，加快建设中华茶祖文化园。继续打好旅游升温战役，编制完善全市旅游产业发展规划，突出“一廊一带”，全面启动云峰湖国际旅游度假区和酒仙湖、东阳湖的建设，加快神农福地、神农城、湘江风光带的建设，积极引进国际品牌酒店，扎实推进炎帝陵创建国家5A级旅游景区，打造知名旅游目的地。积极发展现代物流业，加快南车株洲物流基地、湘江金属物流城、芦淞服饰仓储配送中心、茶陵湘赣物流中心建设步伐。加强房地产市场监管，促进房地产业稳定健康发展。支持金融保险、信息咨询、电子商务、文化创意、服务外包等新型服务业态迅速发展。

　　壮大产业规模。继续实施“5115”工程，支持南车株机、南车株洲研究所、南车电机、株硬、唐人神等企业做大做强。加快推进时代电气IGBT产业化、南车株机扩能二期、通用机场、中航高精传动、山河智能、北汽集团株洲二工厂、南车时代新能源客车基地、中国少儿时尚创意总部基地、攸县煤电一体化、株洲县风力发电、醴陵陶瓷艺术城等项目建设，增强产业发展后劲。深入打好园区攻坚战役，推动产业向园区集中，加大园区基础设施建设力度，形成各具产业特色的园区效应，不断壮大轨道交通、汽车、航空、服饰、陶瓷五大产业集群。实施支持中小企业发展“5115”工程，完善中小企业服务体系，加强产业孵化平台建设，组建互联互保产业联盟，规划建设市中小企业园，培育一批销售收入过5亿元、过10亿元的中小企业。

　　提升产业素质。大力支持企业自主创新，推进技术改造、兼并重组、名牌创建，加强知识产权保护，加快科研成果转化和产业化。重点抓好新能源、新材料技术攻关，提升风电、电动汽车等能源设备制造水平。发展高新技术企业20家以上，高新技术产业增加值占GDP的比重提高到23%，加快建设国家创新型城市。引导优势企业进入资本市场，支持一批中小企业上市。组织“市长质量奖”评选，提升质量管理水平。大力发展特色农业、规模农业、高效农业和休闲农业，加快培育龙华畜牧等一批种养殖业示范基地，支持炎陵白鹅、攸县麻鸭和好棒美做大做强，不断提升农业现代化水平。加大招商引资力度，继续开展“大招商、招大商”活动，围绕五大千亿产业集群和生物医药、新能源、新材料、现代服务业等战略性新兴产业，突出多元化招商、市场主体招商、重点项目招商，大力承接产业转移，积极引进战略投资者，进一步提高利用外资的质量和水平。

　　（二）突出示范引领，推进“两型”建设

　　加快示范区建设。云龙示范区坚持以高端服务业为主导，抓紧建成华强文化科技产业基地和方特梦幻王国；全面启动云峰湖体育公园、龙母河水系水利及景观工程、科技数码城、总部经济园、水利科技园等项目建设。天易示范区加快推进栗雨中央商务区、物流园、汽配园等项目建设，争取中建五局光伏幕墙项目、湘煤立达煤机装备制造建成投产，打造“两型”产业聚集区。积极推进清水塘工业区战略性改造，启动重化工企业“绿色搬迁”，推动传统产业升级换代，抓好清水塘重金属污染治理，加快发展新材料精深加工产业和先进环保制造业，努力把示范区打造成为转变发展方式的引领区。

　　深化“两型”改革。大力推进统筹城乡发展改革，促进基础设施一体化、公共服务均等化、产业发展集聚化、社会保障同城化、社会管理社区化、土地利用集约化。加快投融资体制改革，进一步增强“6+2”投融资公司的市场意识和风险防控意识，加大项目策划力度，提升经营能力；创新投融资模式，探索创建“两型”基金，引导社会资金投资城市建设和产业发展。深入推进城区建设体制改革，发挥区级政府属地管理优势，增强区级政府基础设施建设能力。加快推进土地管理体制改革，提升土地资源配置效率。开展资源节约和环境保护体制改革，创新循环经济发展机制和节能减排实施机制，建立能源差别价格和能耗公示制度，着力推进排污权交易试点工作。

　　构建“两型”环境。坚持“以绿为美、以水为源、以人为本”，围绕“水清、堤固、路畅、景美”的目标，强力推进“一江四港”整治，重点抓好湘江综合整治，加大清障清淤、截污治污、河道采砂整治力度，积极发展沿江生态林、景观林，全面实现沿江企业污水达标排放；启动河东湘江风光带和沿港风光带建设，保护水环境，修复水生态，致力打造“东方莱茵河”。推进节能节水节地节材，提高资源利用效率。加快淘汰落后产能，深入实施节能减排“三百工程”，加快“两型”企业创建步伐。大力发展循环经济，促进资源循环利用、再生利用产业化。稳步推进节水型城市创建工作。深入开展城乡环境同治，全面改善农村环境。

　　（三）突出城镇带动，构建绿色家园

　　拓宽城镇发展空间。坚持规划引领，立足“两型”理念、国际视野、战略思维，科学编制城乡一体的城镇发展规划，积极推进经济社会发展规划、土地利用总体规划、城市总体规划“三规统一”。主城区按照“以现代工业文明为特征的生态宜居城市”的发展定位，坚持“内提外拓、统筹发展”，大力推进云龙新城、武广新城、栗雨新城、新马新城、轨道交通新城、枫溪新城、航空服饰东部新城、金山新城、湘江新城、清水湖生态工业新城等十大新城建设，努力构建“一体三极、三环七射”的城市发展格局。突出抓好县城和中心镇建设，提高县城在县域经济中的首位度，增强中心镇的辐射带动效应，打造一批工业强镇、商贸重镇、文化古镇、旅游名镇。

　　完善城镇基础设施。重点建设神农大道、荷塘大道、航空大道、田心大道等主干道路，建成云龙大道、湘芸中路、泰山西路，启动北环路、沿江路建设，不断完善城区交通网。扎实推进衡茶吉铁路、沪昆高铁株洲段及醴陵站场和长株潭城际铁路等重大基础设施建设。力争建成浏醴、炎睦、分炎高速，加快建设醴茶、炎汝、茶界高速。全面推进G106茶陵县城至炎陵县分路口、炎陵旅游环线、S315攸县县城至分水坳、S320茶陵至界首、S313渌口至谭家山、云龙楠山铺至醴陵塘坊、炎陵沔渡至船形、茶陵和吕至攸县高和等8条干线公路建设。突出抓好通用机场建设。加快建设铜塘湾港区、湘江航道疏浚工程等水运设施。完成市区地下管线普查及信息化一期工程建设，启动城区8处排渍站新建和改造工程，大力提升市区防洪排渍能力。启动“城区直饮水工程五年行动计划”和第二水源建设。积极推进城乡基础设施一体化，新建农村公路200公里，建成农村客运站8座，启动村村通公交试点工程；完成67座病险水库治理，抓好水利灌溉设施扩容改造建设；加快茶陵县、炎陵县扶贫连片开发，着力改变基础设施建设滞后局面；提升云田、松西子、荷塘月色等新农村示范片建设水平，促进城乡互动发展，共同繁荣。

　　提升城镇品质品位。继续推进“四创四化”，大力实施立体绿化、楼顶美化、小街小巷路灯亮化和老旧小区改造，提质改造排水干渠等市政设施，抓好户外广告提质减量，完善全天候、全覆盖的城区保洁网络。提升公共自行车租赁系统建设、管理和经营水平，输出管理模式和产品，打造株洲品牌。牢固树立“大城管”理念，健全运营市场化、管理网格化、作业精细化、考核标准化的城镇管理体系。深入整治城乡结合部、铁路进城沿线、农贸市场、小街小巷等薄弱部位环境卫生。积极开展卫生县城、卫生村镇、文明卫生单位等创建活动。巩固国家卫生城市、全国交通管理模范城市创建成果，加快创建国家环保模范城市、全国文明城市、国家森林城市、国家健康城市。

　　（四）突出科教先导，建设智慧城市

　　提升科技创新能力。充分利用人才资源，打造株洲国家高新技术产业开发区、湖南工业大学科技城和株洲职教大学城三个科教先导区，构建以企业为主体、市场为导向、产学研结合的技术创新体系，建设一批有特色、高水平的实验室和研发中心。大力支持轨道交通装备、中小航空发动机、硬质合金、高分子材料、新能源汽车、半导体、风电装备、陶瓷等产业延伸技术链，完善产业链。加速推进创新成果产权化，产权成果产业化，形成一批拥有自主知识产权、市场竞争力强的高端品牌和产品。

　　提升现代教育水平。深入实施“快乐德育、人文智育、阳光体育”，按照均衡发展义务教育，优先发展职业教育，重点发展高等教育，统筹发展学前、成人和特殊教育的基本要求，抓紧实施《株洲市建设教育强市行动计划》和《株洲市城区基础教育三年攻坚计划》，全面提升教育发展水平。着力提高学前教育普及水平，力争普惠制幼儿园达到30%。加快建宁国际实验学校建设步伐，积极开展义务教育均衡发展示范县（市）区创建活动，规范社会力量办学，大力推进素质教育，促进学生全面发展。加快职教大学城建设，基本建成湖南铁路科技职业学院等5所院校，致力打造中部地区职业教育创新之都。全力支持湖南工业大学新校区建设。

　　提升信息应用能力。加快“智慧株洲”IDC数据中心云平台建设，打造集“技术服务、资源服务、管理服务”三位一体的电子政务建设和管理模式，逐步把政务服务、应急指挥、地下管网、智能交通、市民卡等信息化系统统一到中心平台上进行管理和展示。加快“两化融合”进程，开展万家数字企业创建活动，促进信息技术在产业发展中的推广应用。推进“三网融合”，深化无线网络基础设施建设，构建智能融合的网络设施。启动联网直报指挥中心建设，提高统计工作信息化水平。推进全市协同及移动办公、医疗卫生、“数字食安”监管及公共资源交易等四大平台建设，加快实现各部门信息共享、集约建设，建立新型政府管理服务模式。

　　（五）突出民生优先，提高幸福指数

　　继续完善保障体系。全面落实创业扶持政策，继续实施大学生创业引领计划，深入开展国家创业型城市创建工作；进一步加大职业技能培训力度，积极开发社会公益性岗位，建立城乡统筹的创业、就业服务体系。加快推进收入倍增工程，健全企业、机关和事业单位职工工资增长机制。加大农村剩余劳动力转移力度，切实保障农民工权益，不断提高农民收入。健全城乡社会保障体系，力争城镇养老保险、城镇医疗保险覆盖率达到100%。加强保障性住房建设，启动市区“荷花家园”二期廉租房和荷塘区水竹湖等9个公租房项目建设，加速县市保障性住房建设，加快棚户区和城中村改造步伐，持续推进非公组织公积金制度建设，提高城市居民住房水平。

　　大力发展社会事业。深入推进文化体制改革，健全文化市场监管体系，提高文化市场管理水平。加快发展文化创意、数字出版、影视制作、动漫游戏、会展等文化产业，构建现代文化产业体系。加快农村数字电视工程建设，开拓农村文化市场，推进城乡文化一体化发展。加大公共文化事业投入，建成神农大剧院、神农艺术中心，启动文化园提质改造和青少年活动中心、妇女儿童活动中心、夕阳红文化艺术中心、图书馆、科技馆建设，新建一批示范性乡镇（街道）综合文化站，继续推进农家书屋工程，丰富群众文化生活。发展公共体育事业，广泛开展全民健身和爱国卫生运动，提高群众健康水平。深化医药卫生体制改革，加大公立医院改革试点工作力度，优化公立医院布局，加快卫生信息化建设，提升基层医疗服务能力，完善基本药物制度，为群众提供优质高效的医疗服务。加强人口计生工作，增强可持续发展能力。发展社会福利、慈善、法律援助等社会公益事业，维护残疾人和妇女儿童权益。做好民族宗教、对台、市志、档案等工作。

　　切实维护社会稳定。高度重视信访维稳工作，抓好市长热线等平台建设，及时化解各类矛盾纠纷。加强国防教育、人民武装和国家安全工作。狠抓重点区域消防隐患整治，强化安全生产监管，加强公共安全体系建设，完善突发事件预测预警机制和应急处置机制。加快建设食品安全检验检测体系，努力实现从田间到餐桌的全程监管。加强价格调控，保持物价基本稳定。深入推进平安株洲建设，加大公安“一化三基”正规化建设力度，积极预防和严厉打击各类违法犯罪活动，有力保障广大人民群众的生命财产安全。

　　强化管理：着力提升服务发展水平

　　实现今年的目标任务，我们必须坚持激情工作，规范管理，勤政廉政，依法行政，努力为经济社会又好又快发展提供坚强保障。

　　创新社会管理。完善源头治理、动态管理和应急处置相结合的社会管理机制，全面提高社会管理科学化水平。创新流动人口服务管理工作，推行流动人口“一证通”制度，着力解决流动人口就业、居住、就医、子女就学等困难。加强城乡社区建设，增强社区自治和服务功能，加快推进“政居分设、选聘结合”的社区管理体制改革，把城乡社区建设成为管理有序、服务完善、文明祥和的社会生活共同体。引导社会组织参与公共服务和社会管理，积极推进政府向社会组织购买服务。健全调解组织，完善专业调处机制，构建大调解格局，努力化解各类矛盾纠纷。

　　强化要素保障。加大各类人才的培养、引进和使用力度，启动千名海外人才集聚工程，做好海外留学人才引进工作，培养一大批创新型人才和高技能人才队伍。制定科学有效的水、电、气等资源供应方案，保障生产生活能源需求。强化用地管理，推进集约节约用地，加大土地征迁工作力度，确保土地有效供给。加强资金保障，充分发挥财政资金引导作用，强化银行信贷资金支撑作用，拉动企业和民间投资，推动股权类投资机构加大投资力度，保障战略性新兴产业发展及重点项目建设资金需求。

　　优化发展环境。全面推进政务公开，加快各级政务服务中心规范化建设，继续推进行政许可网上审批及电子监察系统建设，深入推进市级建设工程联合验收工作，完善项目审批快速办理机制，营造廉洁高效的政务环境。大力弘扬“火车头精神”，推动城市精神与经济社会发展的融合，营造开放包容的人文环境。积极推进基层民主法治建设，畅通诉求渠道，加强法治宣传，营造民主公正的法治环境。规范市场经济秩序，加强诚信和职业道德教育，构建社会信用体系，营造公平竞争的市场环境。

　　加强政府自身建设。努力提高行政效能，创新管理方式，推动行政管理流程再造，完善科学民主的决策机制、精简效能的执行机制、奖罚分明的激励机制和权责统一的问责机制，科学设置政府绩效考核指标和考核办法，强化行政效能监察，确保政令畅通，确保各项工作任务落实到位。坚持依法行政，全面落实《法治湖南建设纲要》，自觉接受人大及其常委会的监督，坚决执行人大及其常委会的决议、决定，积极支持人民政协参政议政，认真办理人大代表建议和政协委员提案。严格落实党风廉政建设责任制，健全反腐倡廉长效机制，不断提高政府公信力和执行力。坚持勤俭办事，用制度管权管人管事，大力倡导艰苦奋斗的良好作风，努力打造一支清正廉洁、务实高效、作风过硬的公务员队伍。

　　各位代表，“四个株洲”，其势已成；“三个加快”，其时已至。让我们在市委的坚强领导下，认真贯彻落实市第十一次党代会精神，同心同德，砥砺奋进，心无旁骛办实事，身体力行抓落实，为建设智慧株洲、实力株洲、绿色株洲、幸福株洲而努力奋斗 ！
